# Rethinking Scientific Summarization Evaluation: Grounding Explainable Metrics on Facet-aware Benchmark

**Published:** 2024-02-22

**Authors:** Xiuying Chen, Tairan Wang, Qingqing Zhu, Taicheng Guo, Shen Gao, Zhiyong Lu, Xin Gao, Xiangliang Zhang

**Affiliations:** 1 King Abdullah University of Science & Technology; 2 National Center for Biotechnology Information, National Library of Medicine, National Institutes of Health, Bethesda, MD, USA; 3 University of Notre Dame; 4 Shandong University

## Abstract

The summarization capabilities of pretrained and large language models (LLMs) have been widely validated in general areas, but their use in scientific corpus, which involves complex sentences and specialized knowledge, has been less assessed. This paper presents conceptual and experimental analyses of scientific summarization, highlighting the inadequacies of traditional evaluation methods, such as *n*-gram, embedding comparison, and QA, particularly in providing explanations, grasping scientific concepts, or identifying key content. Subsequently, we introduce the Facet-aware Metric (FM), employing LLMs for advanced semantic matching to evaluate summaries based on different aspects. This facet-aware approach offers a thorough evaluation of abstracts by decomposing the evaluation task into simpler subtasks. Recognizing the absence of an evaluation benchmark in this domain, we curate a Facet-based scientific summarization Dataset (FD) with facet-level annotations. Our findings confirm that FM offers a more logical approach to evaluating scientific summaries. In addition, fine-tuned smaller models can compete with LLMs in scientific contexts, while LLMs have limitations in learning from in-context information in scientific domains. This suggests an area for future enhancement of LLMs^1^.

## 1 Introduction

Scientific summarization aims to distill the primary content of scientific papers into brief abstracts, often structured around background, method, results, and conclusion (Wang et al., 2023). Given the specialized nature and critical need for accurately representing scientific findings, numerous summarization datasets spanning from medicine to computer science have been presented (Cohan et al., 2018), accompanied by models with dedicated architectures (An et al., 2021; Zhang et al., 2022; Koh et al., 2022). However, the assessment of scientific summarization is less studied and often depends on traditional text generation metrics (e.g., ROUGE, BERTScore), despite its unique requirements. A scientific summary must be assessed for its comprehensibility and accuracy in reflecting the paper’s core content, to save readers time and prevent misunderstanding or misleading information.

**Figure 1:**
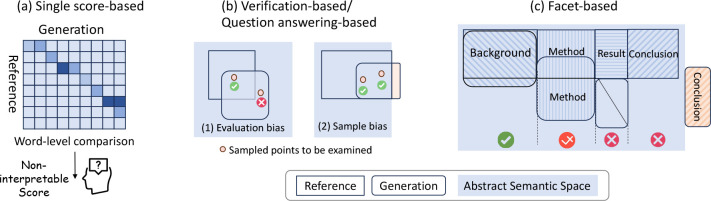
(a) Single score methods like ROUGE and BERTScore lack explainability. (b) Verification methods like ACU show *evaluation bias* against correct non-reference claims and *sample bias* in semantic coverage. (c) FM metric employs LLMs for broader semantic matching and splits evaluation into clear and consistent facets. The *abstract semantic space* denotes all the semantics that can be found or inferred from the reference.

Our analysis reveals that single-score methods like ROUGE (Lin, 2004) and BERTScore (Zhang et al., 2020) mainly focus on word-level comparisons between the reference and generated summary, overlooking the subtleties of semantic understanding and lacking interpretable reasoning. QA-based and verification methods, such as QuestEval (Scialom et al., 2021) and ACU (Liu et al., 2023a), compare the generation with reference by sampling limited units from the continuous semantic space, limiting their ability to conduct a thorough assessment. For example, reference may not fully cover all the correct information, and units sampled outside the reference but within the semantic space do not necessarily indicate inaccuracy (evaluation bias in Fig. 1(b)). In another scenario (sample bias in Fig. 1(b)), errors might go unnoticed if no samples are taken from outside the semantic space for evaluation.

In this paper, we address the above challenges by introducing a novel Facet-aware Metric (FM), which leverages LLMs’ broad knowledge base and sophisticated semantic matching abilities for evaluating scientific summary. Inspired by the structured abstract format (Sollaci and Pereira, 2004), we partition the abstract into four distinct sections: background, method, result, and conclusion, and then compare the generated abstract with the original across each of these segments, as shown in Fig. 1(c). The benefits of our metric are threefold: 1) It performs continuous semantic matching instead of breaking the semantics into discrete points, mitigating the two biases previously introduced. 2) It emphasizes the role of each segment, enabling a clearer understanding of the paper’s core concepts and finer-grained explanation (Fok et al., 2023). 3) Breaking down the evaluation into more specific criteria reduces the complexity of the task and minimizes inconsistencies among different annotations.

To showcase the effectiveness of FM and foster further research into evaluation methods for scientific summarization, we created FD, a benchmark designed to facilitate the comparison of different evaluation metrics. FD comprises 500 abstracts generated for 100 papers across various domains from PubMed and arXiv. The quality of these abstracts, produced by different models, exhibits a wide range of deficiencies. Human annotations were meticulously constructed to identify and highlight their issues across different aspects. We consider this benchmark a significant contribution to the advancement of evaluation methods for scientific summarization.

Upon a thorough quality analysis of abstracts in FD using existing metrics, we observed consistent discrepancies between existing automated metrics and human evaluations. In contrast, our FM metric provides profound interpretability at both the granular and overall summary levels, aligning closely with human evaluations. Lastly, our research uncovers insightful findings shown in Tab. 1, highlighting directions for enhancing the scientific summarizaion performance.

## 2 Related Work

### Summarization on Scholar Papers.

Automatic summarization for scientific papers has been studied for decades, with earlier research emphasizing document content and favoring extractive methods (Cohan and Goharian, 2018; Xiao and Carenini, 2019). Recently, abstractive models have demonstrated enhanced effectiveness in summarizing scholarly texts. Specifically addressing the challenges of summarizing long documents, BigBird (Zaheer et al., 2020), employs a sparse attention mechanism that effectively reduces the quadratic dependency to linear for longer sequences. Furthermore, LongT5 (Guo et al., 2022) incorporates attention mechanisms suited for long inputs and integrates pre-training strategies from summarization into the scalable T5 architecture. More recently, LLMs such as Llama (Touvron et al., 2023) have also achieved notable performance in this domain.

### Automatic Evaluation Metrics.

To evaluate the performance of summarization models, numerous metrics have been proposed to compare the generated summary against the ground truth reference. Earlier metrics such as ROUGE (Lin, 2004), METEOR (Banerjee and Lavie, 2005), and SERA (Cohan and Goharian, 2016) primarily utilized overlapping *n*-gram calculations. With advancements in pretrained language models, embedding-based metrics like BERTScore (Zhang et al., 2020) and BARTSCORE (Yuan et al., 2021) emerged, which are based on vector calculations but lack intuitive explanations. Later, Scialom et al. (2021); Kryściński et al. (2020); Fabbri et al. (2022); Tam et al. (2022) introduce aspect-aware metrics, predominantly focusing on faithfulness through a question-answering paradigm. Most recently, Liu et al. (2023b) propose to extract units from one text and subsequently verify it against another. However, this metric struggles in the scholarly domain, possibly due to the difficulty of extracting semantic units from complex academic texts.

### Human Evaluation Paradigm.

To assess automatic evaluation metrics, it is essential to compare them against human evaluation scores. Traditional human evaluation methods involve assigning an overall faithfulness or consistency score to the generated summary (Gao et al., 2019; Fabbri et al., 2021), or utilizing pairwise comparisons (Chen et al., 2018). Recent studies aim to provide a more nuanced assessment of progress in summarization models and metrics. Some important efforts focus on general domain (Bhandari et al., 2020; Liu et al., 2023a), which annotates summaries according to semantic content units, a semantic unit motivated by LitePyramid protocols (Shapira et al., 2019). However, our experiments reveal that dissecting information in the scholarly corpus is challenging due to its dense content and often more intricate grammar compared to the general domain.

## 3 Facet-based Evaluation Metric

### 3.1 Rethinking on Existing Metrics

#### Single-Score Metric.

Traditional metrics, ROUGE and BERTScore, are central in evaluating NLP-generated texts. ROUGE emphasizes recall by analyzing *n*-gram overlaps between reference and generated summaries, while BERTScore measures pairwise word-level similarity using representations computed by pre-trained language models RoBERTa (Liu et al., 2019). However, these metrics fail to capture the continuous semantic meaning of text, instead reducing it to word-level comparisons. Furthermore, they only yield single scores without providing details on attributes, such as where and why errors occur, reducing their trustworthiness and reliability.

#### Question-answering based Metric.

Recent explainable evaluation metrics, like QuestEval (Scialom et al., 2021), use question-answering paradigms to assess the accuracy of generated summaries. These methods focus on entities and nouns as answers, formulating questions to compare answers derived from both the reference and the generated summary. While this method has a relatively higher correlation with human evaluations, we summarize its limitations as follows.

**Figure 2:**
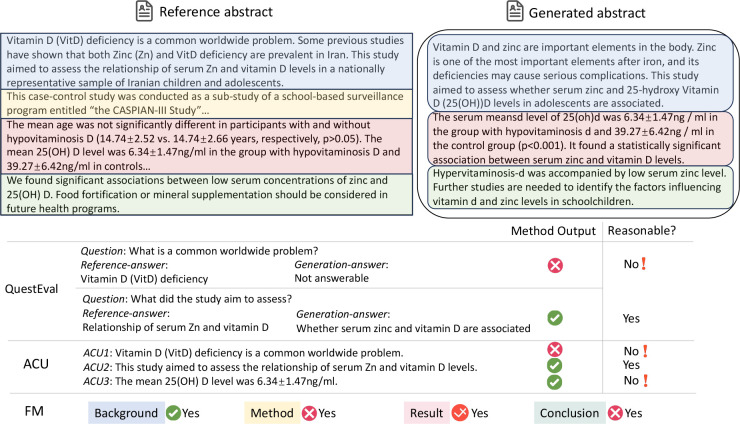
An evaluation case study on PubMed dataset. QuestEval and ACU require precise alignment between the reference and generated text, which is often unachievable in real-world scenarios. The semantic segment under scrutiny may not cover the entire semantics of the text either. Conversely, our FM metric enables holistic semantic evaluation without segmentation and eases the process by semantic matching across different facets.

Firstly, relying solely on a limited set of QA pairs for evaluation risks *sample bias*. This is because the meaning of a text is not merely a compilation of isolated facts or data points. Rather, it is an interconnected continuum, where ideas, concepts, and nuances interweave. A limited number of samples might not fully capture this continuum of meaning. Moreover, discrepancies in answers to a question between the reference and generated summaries do not necessarily indicate that the generated summary is incorrect, which we name as *evaluation bias*. For example, as illustrated in Fig. 2, the generated content fails to directly answer the question and highlight that “Vitamin D deficiency is a common worldwide problem”. However, the generation also notes the importance of Vitamin D, thereby providing a relevant and informative background introduction. Nevertheless, according to the QA rule, this would be erroneously labeled as unfaithful content.

#### Claim Verification based Metric.

In a related line of work, the evaluation of summaries is conducted by verifying their claims. For instance, Kryściński et al. (2020) extract specific spans from the reference text to assess their consistency with the generated content. Building on this, Liu et al. (2023b) introduce ACU, a method that replaces spans with atomic content units, as they find that using smaller annotation units improves annotation consistency. This technique addresses the previous challenge of formulating appropriate questions for examination.

Yet, the two previous biases still exist. First, comprehensively covering all points in a sentence, especially in scientific texts characterized by complex structures and specialized terminology, is challenging. For instance, consider the reference in Fig. 2, which includes intricate concepts such as “VitD”, “Zinc”, “25-hydroxy”, and “cardiometabolic”, along with their relationships. Such sentences present a more complicated scenario for segmentation, particularly when compared to the simpler and more straightforward structures in general domains like news. Secondly, various methods exist to convey similar meanings; however, examining at the unit level necessitates precise alignment, which is not always feasible in practical scenarios. Thirdly, the accuracy of a unit is context-dependent. For instance, consider the ACU3 in Fig. 2. Without knowing the group being examined, it’s uncertain whether the claim holds true.

We also tried the recent TIGERSCORE (Jiang et al., 2023), which is designed to generate evaluation scores with explanations in a sequence-to-sequence approach. Unfortunately, this model struggles to produce fluent sentences and fails to yield scores on scholar corpus. This limitation likely stems from its heavy reliance on its training corpus, which does not include scholarly texts.

#### Domain Specific Metrics.

To meet the specialized needs of medical reviews, distinct strategies have been proposed. For example, Huang et al. (2006) propose a PIO framework, which classifies systematic reviews based on three aspects: *P**opulation*, *I**ntervention*, and *O**utcome*. Based on this concept, Delta was presented by Wallace et al. (2021) and later refined by DeYoung et al. (2021). This method calculates the probability distributions of evidence direction for all I&O pairs in both the target and generated summaries. The final score is derived by applying the Jensen-Shannon Divergence to compare these distributions for each I&O pair, where a lower score indicates a closer alignment with the target summary. However, abstracts of scientific papers can span various domains, and the PIO components are exclusive to medical reviews.

### 3.2 Facet-based Evaluation Paradigm

Given the aforementioned challenges, we propose a facet-aware evaluation paradigm tailored to the unique attributes of scientific abstracts. Building on the foundational work of (Dernoncourt and Lee, 2017) and (Jin and Szolovits, 2018), which categorized abstract sentences into groups like *Background, Method, Result*, and *Conclusion*, we classify abstract content into distinct facets, forming the BMRC set. Specifically, ‘Background’ includes the introductory background and objectives of the work, ‘Method’ details the experimental methods and comparisons, ‘Result’ covers experimental observations and data analysis, and ‘Conclusion’ encompasses the drawn conclusions, including any limitations or future perspectives of the work. We reviewed papers on PubMed and arXiv, and found that paper abstracts within the fields of biomedical sciences, physics, and computer science generally follow the BMRC structure. We are also aware that not all abstracts adhere to the BMRC structure. For example, a survey paper may not include a method section. In such cases, we remove the corresponding aspect in the evaluation.

For a quantitative assessment of the alignment between the reference (Input1) and generated abstracts (Input2), they are compared on the *Background* and *Conclusion* facets based on the following rating rules:

- 3: Input2 is generally consistent with Input1.

- 2: Input1 is not mentioned in Input2.

- 1: Input2 contradicts Input1, or Input2 lacks relevant content in this aspect.

Take the *background* shown in Fig. 2 for example, both the reference and generated text emphasize the importance of Vitamin D and Zn in the human body. Consequently, the generated summary receives a score of 3 regarding *background*. In contrast, the generated *conclusion* inaccurately claims ‘hypervitaminosis-d is accompanied by low zinc level’, whereas it should state ‘hypovitaminosis’. This error resulted in a lower score of 1.

The rating rule for evaluating *Method*/*Result* is:

- 4: Input2 generally includes Input1’s information, or omits minor details from Input1.

- 3: Input2 generally includes Input1’s information, but omits a part of the key information from Input1.

- 2: Input2 is not empty, but it does not mention any key information in Input1.

- 1: Input2 contradicts Input1, or Input2 lacks relevant content in this aspect.

Here, ‘key information’ comprises the essential elements crucial for understanding the core message of Input1, while ‘minor details’ are less critical supplementary elements whose omission doesn’t significantly alter the overall understanding. Take the case in Fig. 2 for example, the generated *result* section indicates a correlation between Zn and Vitamin D, but it omits whether this relationship is positive or negative. This leads to a score of 3, as the direction of the relationship (positive) is vital for fully understanding the conclusions of the study. We use a 3-point scale for general *background* and conclusion sections and a 4-point scale for detailed *method* and *results* sections to capture nuances. We are aware that different abstracts may highlight various key information. Here we regard the paper’s abstract as ground truth following other evaluation works (Liu et al., 2023a), leaving multi-reference evaluation to future research.

Based on the rating score of each aspect, the overall score of a generated abstract is as follows, with the weight of each aspect as introduced in detail in §4.2.

(1) $$s=(\sum_{i=1}^{4}\;{\text{score}}_{i}/{\text{scale}}_{i}\times {\text{weight}}_{i})/4.$$

We will construct a human annotation benchmark dataset following this paradigm in §4.

### 3.3 LLM-based Facet-aware Evaluation

Given the proficiency of LLMs in text comprehension, we can utilize them to automatically assign facet-aware scores following our facet-based evaluation paradigm. Due to the intricacies of comprehending scholarly corpora and to simplify the task, we divide the assessment into two sub-tasks: first, LLM extracts facet-aware segments from both reference and generated abstracts. Next, the segments are compared using LLMs, guided by prompts in §4, and a weighted sum is applied to calculate the final score as in Eq. 1. Compared to prior evaluation metrics, our evaluation paradigm offers both transparency and insight into the scores produced, while also considering various facets of an abstract, reflecting its role in the user’s reading experience.

We utilize GPT-3.5, GPT-4, and Llama2 as the foundational LLM for our tasks, denoted as FM (backbone_name). We also compare with other variations such as:

FM (backbone w/ few): We keep the decomposition step but add random few-shot examples, to see the contribution of in-context learning. The examples can be found in Appendix A.1.

Vanilla backbone: The LLM is directly fed with rating instructions ‘*Rate the alignment between the two inputs on a scale from 1 (worst) to 4 (best)*’, bypassing the decomposition process.

We will show the effectiveness of LLM based on our FM paradigm in §5.2.

## 4 Facet-based Evaluation Benchmark

To the best of our knowledge, there is currently no evaluation benchmark for the scientific paper summarization domain. Based on our proposed facet-aware evaluation paradigm, we introduce a facet-based evaluation dataset, which will be used to assess automatic evaluation metrics.

### 4.1 Summarization Systems

We construct our benchmark based on two datasets: arXiv and PubMed. While arXiv predominantly contains papers from fields such as physics, mathematics, and computer science, PubMed is centered around biomedical literature. We randomly sampled 50 cases from arXiv for evaluation. For PubMed, we utilize the test set produced by Krishna et al. (2023), comprising 50 cases.

For each paper in the arXiv dataset, we select the pretrained summarization model BART-large (Lewis et al., 2020), and the recent state-of-the-art model Factsum (Fonseca et al., 2022). Additionally, we incorporate abstracts generated by leading LLMs, specifically Llama2–70b (Touvron et al., 2023), GPT-3.5 and GPT-3.5 w/ few-shot learning. On each paper in the PubMed dataset, we utilize pretrained models BigBird-PEGASUS-large (Zaheer et al., 2020) and LongT5-large (Guo et al., 2022) as recommended by (Krishna et al., 2023). We also include the ‘block’ version of LongT5 and BigBird that prevents 6-grams from being directly copied from the source to reduce the extractiveness. In total, our benchmark comprises 500 abstracts generated by different summarization systems.

### 4.2 Human Evaluation Process

We have two annotators, who are PhDs with expertise in both bioinformatics and computer science. Together, they select cases to serve as in-context examples for a few-shot learning setting. Subsequently, they independently annotated all cases, evaluating pairs of (target, generated) summaries from a paper based on four facets. Whenever there were differences in their scores, the annotators engaged in discussion to reach a consensus. This annotation process also aligns with previous studies (Jin et al., 2019; Liu et al., 2023a). The inter-annotator agreement, measured by Cohen’s Kappa and agreement proportions for all four facets, is shown in Tab. 3. Notably, the method and result facets showed lower agreement, consistent with the expectation of their varied classification levels. Overall, the agreement rates exceeded those in previous datasets like ACU and medical literature reviews (Wang et al., 2023). Furthermore, to assess the relative importance of different facets in the abstract and compute an overall score, an additional annotation step was conducted. Here, one annotator assigned overall scores to each summary. These scores were used to derive the weights of individual components through linear fitting, resulting in weights of [0.1, 0.3, 0.3, 0.3] and a mean squared error of 0.005, indicating a strong fit.

## 5 Benchmark Analysis

### 5.1 Comparing Summarization Systems

In Tab. 2 we show the performance of the compared summarization systems in different metrics on the PubMed dataset. We do not include GPT-3.5’s with few-shot learning, as it does not improve performance. Similar results on arXiv and other details are in Appendix B. Generally, GPT-3.5, Llama2, and Long5 consistently achieve higher evaluation scores across all metrics, showing their robustness and adaptability in different domains. Specifically, Llama2 shows the highest performance, similar to the observation in the news domain (Kadous, 2023). This highlights the potential of applying open-source LLMs as alternatives to closed LLMs like those of OpenAI. The result also suggests that *finetuned smaller-scale models can rival the performance of LLMs in scientific contexts.* However, achieving such performance demands precise model design, as seen in BigBird’s inferior performance compared to LLMs.

We also present a statistical evaluation in Fig. 4. Our findings indicate that *while GPT-3.5 tends to produce text that is easier to understand, it often misses critical scientific statistics.* As shown in Fig. 4(a), it generates an extended background in the abstract, but includes significantly fewer numbers in its text compared to other models, as seen in Fig. 4(b). This is notable given the importance of numerical data in scientific literature. Additionally, GPT-3.5 favors the use of more commonly used words, a trend that is evident in Fig. 4(c). In addition to the overall evaluation, we detail the human assessment in different facets in Appendix C. Case studies show that when the conclusion diverges from standard background information, GPT-3.5 tends to stick to conventional knowledge rather than aligning with the provided conclusion. Furthermore, our statistical analysis indicates that 34.7% of weaker performances in PLM are linked to fluency challenges in generating lengthier text in final conclusions.

### 5.2 FM Metric Analysis

We next assess the evaluation metrics.

#### Benefits of Our FM.

**Figure 3:**
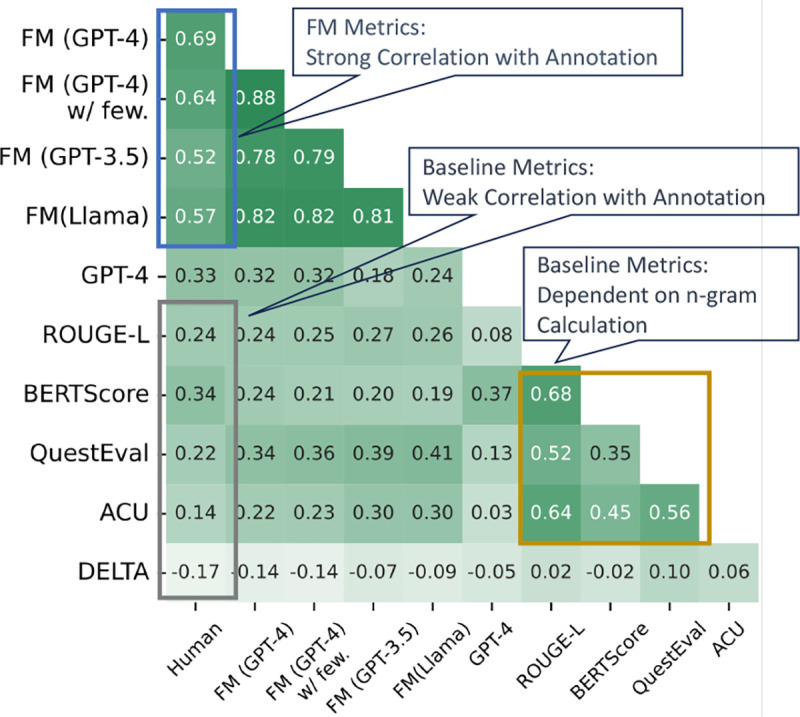
Spearman correlations among metrics within our FM paradigm, LLM-based baseline (GPT-4), and existing evaluation metrics (ROUGE-L, etc).

Firstly, *our decomposed evaluation paradigm simplifies the evaluation task for LLMs*, without requiring advanced reasoning capabilities. As indicated in the blue box in Fig. 3, our FM family metrics consistently exhibit a strong correlation with human evaluations, reaching an impressive correlation of up to 0.69. Conversely, GPT-4 and Llama2 without employing the decomposition strategy, fail to achieve a high correlation. Additionally, to assess the first step’s support for subsequent steps in §3.3, we conducted a human evaluation detailed in Appendix A.2. For example, we discovered that GPT-4 has a 92% accuracy rate, demonstrating its high reliability and solid foundation for subsequent procedures. We also show case study in Fig. 11 in Appendix.

Secondly, *the existing evaluation metrics show a moderate correlation with human scores*, as seen in the gray box of Fig. 3, with correlations below 0.4, and further confirmed by Tab. 2. For example, BERTScore’s similar ratings for different models reveal its limited differentiation capability. Delta also faces challenges, particularly in transitioning from medical reviews to paper summarization (see Tab. 2). TIGERSCORE, not included in the table, consistently fails to produce scores, highlighting the need for robustness and generalizability in embedding-based metrics, especially when applied to specialized domains.

Thirdly, *our approach offers a deeper semantic analysis beyond mere n-gram overlaps.* Traditional metrics like BERTScore, QuestEval, and ACU show a strong correlation with ROUGE-L (yellow box in Fig. 3) and consistently favor LongT5 as the top model (Tab. 2). This suggests that these metrics rely on word sequence overlaps rather than overall understanding. In contrast, metrics following our FM approach like FM (GPT-4), rank Llama2 higher, aligning closely with human evaluations.

#### LLM Analysis.

Unlike traditional approaches that assess LLMs through direct question-answering, our framework employs a meta-evaluation method to examine LLMs’ evaluative capabilities. Firstly our analysis reveals that few-shot learning prompts fail to enhance the performance of both GPT-3.5 and GPT-4. We assume that this is because *the limitation of LLMs lies in lack of ability to learn scientific knowledge through in-context learning*. This hypothesis is further supported by observations that introducing few-shot learning in the summarization process also fails to improve performance. This suggests an area for future enhancement of LLMs.

#### Comparison of QA and Verification based Methods.

When comparing verification-based metrics with question-answering-based metrics, we find that the question-answering paradigm (QuestEval) significantly outperforms the former (ACU) across two datasets, such as in Fig. 3. This suggests that *breaking semantic meanings into units is challenging for language models, whereas answering concise questions with brief phrases and words is more straightforward and effective.*

## 6 Effectiveness of Decomposition

### 6.1 Decomposition in Summary Evaluation

**Figure 4:**
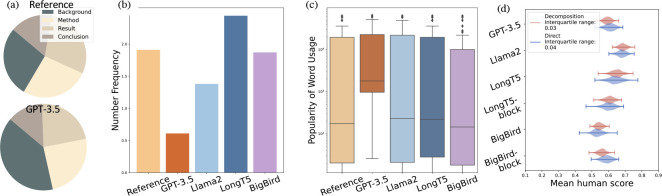
(a) Proportions of different facets. (b) Frequency of numbers in reference and generated text. (c) Popularity of word usage. (d) Mean human evaluation score distributions for various models shown by violin plots, comparing two annotation methods through bootstrap resampling.

In Fig. 3 we show that GPT-4 underperforms our FM (GPT-4) by a large margin, demonstrating the effectiveness of decomposition in automatic evaluation process. Furthermore, in Fig. 4(d), we provide a violin plot comparison of human evaluation scores using decomposition and direct annotation methods. These plots are generated through bootstrap resampling, a robust method for assessing score consistency over multiple evaluations (Krishna et al., 2023; Cohan and Goharian, 2016). *The decomposition method demonstrates a significant advantage in producing reliable and consistent annotations*, as evidenced by its considerably narrower interquartile range (0.26 compared to 0.40).

It is also important to note that despite their methodological differences, both the decomposition and direct annotation methods yield the same relative ordering of systems. This consistency underscores that the model scoring is not dependent upon the specific method used; instead, it remains consistent across different annotation strategies. This further highlights that *the metrics following our paradigm are not just consistent with human evaluation results within the same paradigm, but correlated with the gold standard evaluation.*

### 6.2 Decomposition in Summary Reading

**Figure 10:**
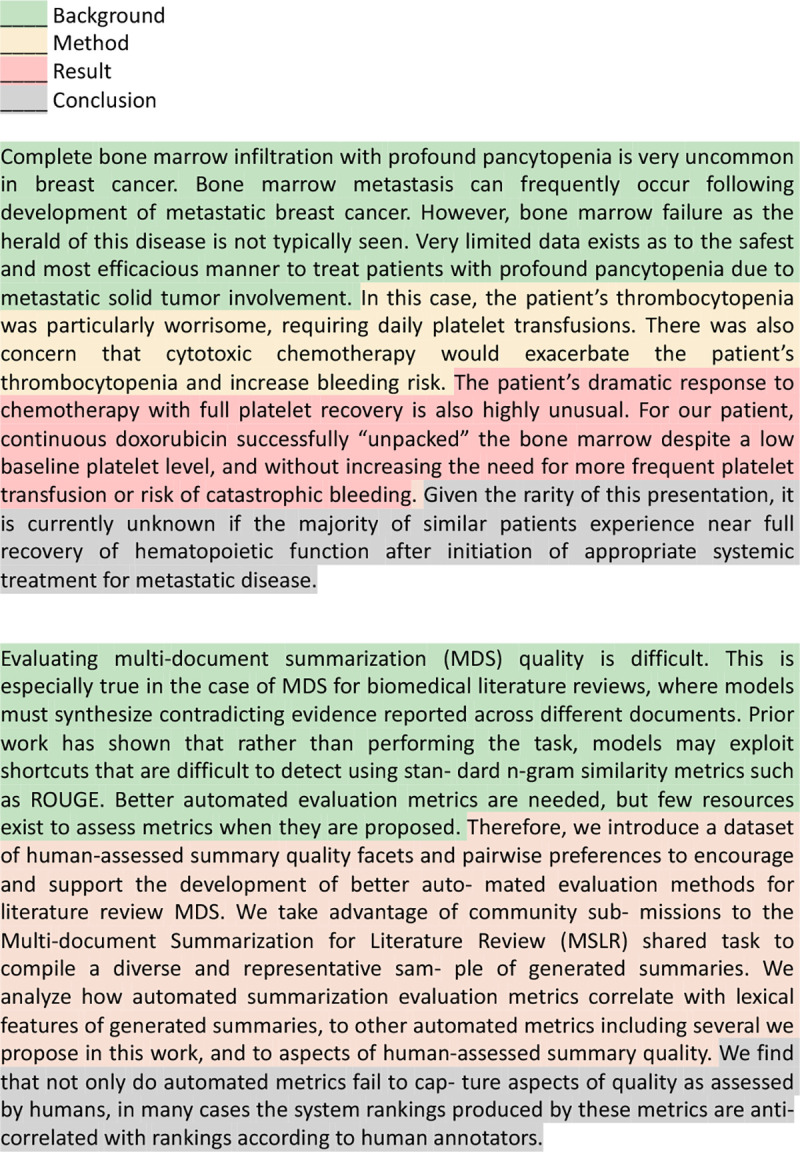
Highlight visualization for reading summaries during the question-answering task.

**Figure 11:**
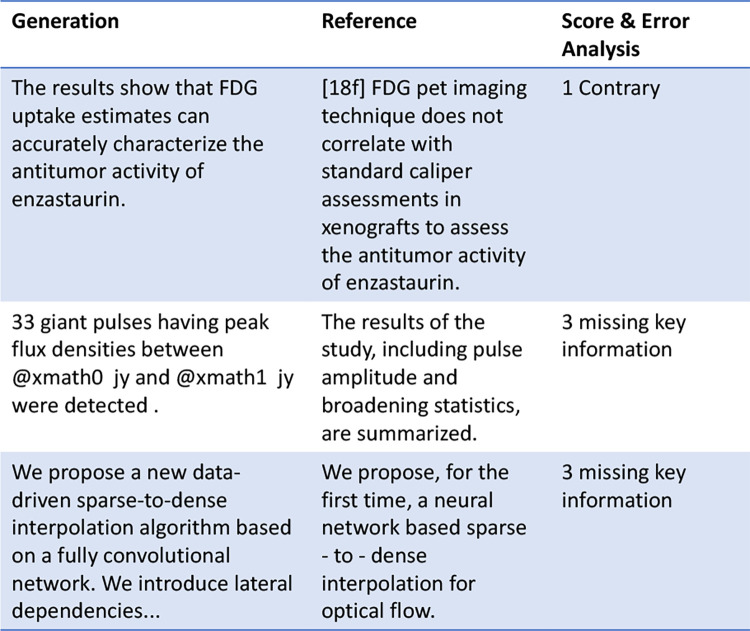
Case study across two datasets of our FM (GPT-4).

Since we obtained the abstract with decomposition markers, we are interested in exploring whether this decomposition aids in the user’s reading process. Concretely, we recruited six PhD participants in reading papers sampled from the two datasets. They skimmed a paper abstract and responded to two multiple-choice questions. We tracked the time taken to answer and the accuracy of the participants’ first responses. Rating interface can be found in Fig. 10 in Appendix. Participants using our markers answered questions faster (average time *μ* = 47.9s, standard deviation *σ* = 20.8s) compared to a standard document reader (*μ* = 55.0s, *σ* = 23.2s), with the difference being statistically significant (*p* < 0.05). Additionally, 4 out of 6 annotators found that decomposition makes the task easier. Notably, this time efficiency did not affect accuracy, as there was no significant difference in accuracy between decomposition (*μ*= 0.82, *σ*= 0.34) and plain text reading (*μ* = 0.79, *σ* =0.31). This demonstrates *decomposition on abstract is also beneficial in reading processes* (Sollaci and Pereira, 2004).

## 7 Conclusion

In this study, we analyze the shortcomings of current summarization evaluation metrics in academic texts, particularly in providing explanations, grasping scientific concepts, or identifying key content. We then propose an automatic, decomposable, and explainable evaluation metric, leveraging LLMs for semantic matching assessments. We also introduce the first benchmark dataset spanning two scholarly domains. Our study highlights significant gaps between automated metrics and human judgment, with our metric aligning more closely with the ground truth. We also uncovered numerous insightful findings for summarization and evaluation of scholar papers. Looking ahead, our future work aims to explore multi-reference or reference-free evaluation techniques in the scientific field.

### Limitation

Our evaluation metrics rely on the presence of reference summaries, primarily due to the existence of accurate and faithful abstracts for scientific papers. Nonetheless, our ultimate goal is to assess summary quality without the need for references. There are existing reference-free summarization evaluation techniques (Gao et al., 2023), but the performance of these metrics in scientific summarization evaluation has yet to be studied, marking an area for future research. Meanwhile, it’s worth noting that a single paper could have several fitting abstracts. While our evaluation criteria take into account the varied ways one might craft a competent abstract, having a broader set of human-composed abstracts as a benchmark would be advantageous. Our approach is flexible enough to work with multiple references, and we plan to explore frequency modulation using various sources in our future research.

### Ethical Consideration

The use of LLMs to evaluate summaries introduces complex ethical considerations. These include the potential for biases encoded within the model to influence assessment outcomes, raising concerns about fairness and equity. Privacy risks emerge from the utilization of sensitive data in LLM training, with implications for consent and confidentiality. Responsible implementation of LLM-based evaluation requires proactive measures to address biases, ensure transparency, obtain informed consent, and mitigate potential harms. Thus, while LLMs offer promising evaluation capabilities, ethical safeguards must be prioritized to uphold fairness, transparency, and respect for individual privacy and autonomy.

## Figures and Tables

**Table 1:** Summary of the key findings in our work.

§5.1 Comparing Summarization Systems:
• Larger is not always better: finetuned smaller models rival LLMs in scientific contexts.
• GPT-3.5 tends to produce text that is easier to understand but often misses critical scientific statistics.
§5.2 Comparing Evaluation Metrics:
• Existing evaluation metrics show a moderate correlation with human scores and a high inter-correlation with ROUGE scores, emphasizing *n*-gram overlap.
• Our decomposed evaluation paradigm simplifies and excels, moving beyond mere *n*-gram calculation.
• LLMs have limitations in learning from in-context information in the scientific domain.
• Decomposition is beneficial for both evaluating and understanding abstracts.

**Table 2:** Performance of various summarization systems in different metrics. 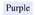 cells indicate the best result, while 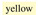 cells denote the second best. In general, the smaller pretrained LongT5 competes well with Llama2 across different metrics. Specifically, FM-based methods tend to favor Llama2, in contrast to existing metrics that primarily rely on *n*-gram overlap calculations similar to ROUGE.

Model	ROUGE-L	BERTScore	DELTA	QuestEval	ACU	FM(Llama2)	FM(GPT-3.5)	FM(GPT-4)	Human

GPT-3.5	0.2109 (6)	0.8408 (2)	0.4512 (2)	0.2333 (6)	0.1799 (3)	0.7691(3)	0.6343 (4)	0.6623 (4)	0.6780 (4)
Llama2	0.2223 (4)	0.8408 (2)	0.4629 (1)	0.2678 (2)	0.1835 (2)	0.8769(1)	0.7228 (1)	0.7120 (1)	0.7704 (1)
LongT5	0.2832 (1)	0.8534 (1)	0.4106 (5)	0.2699 (1)	0.2161 (1)	0.7719(2)	0.6591 (2)	0.6818 (2)	0.7241 (2)
LongT5-block	0.2345 (2)	0.8408 (2)	0.4113 (4)	0.2496 (3)	0.1524 (4)	0.7207(5)	0.6283 (5)	0.6628 (3)	0.6785 (3)
BigBird	0.2240 (3)	0.8317 (6)	0.4432 (3)	0.2376 (5)	0.1405 (5)	0.6186(6)	0.5947 (6)	0.5649 (6)	0.6186 (6)
BigBird-block	0.2127 (5)	0.8383 (5)	0.3891 (6)	0.2392 (4)	0.1222 (6)	0.7347(4)	0.6475 (3)	0.6167 (5)	0.6317 (5)

**Table 3:** Inter-annotator agreement between experts on facets (Cohen’s *κ* and proportion of agreement).

Facet	Classes	*κ*	Agreement

Background	3	0.91	0.83
Method	4	0.78	0.69
Result	4	0.86	0.79
Conclusion	3	0.90	0.85

**Figure 5:**
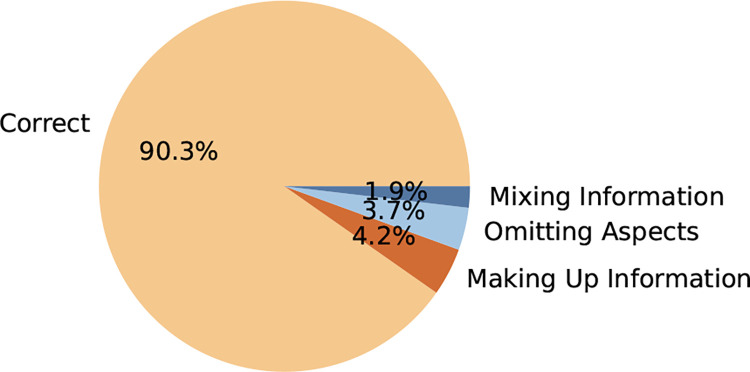
Human evaluation of GPT-4’s facet information extraction.

**Figure 6:**
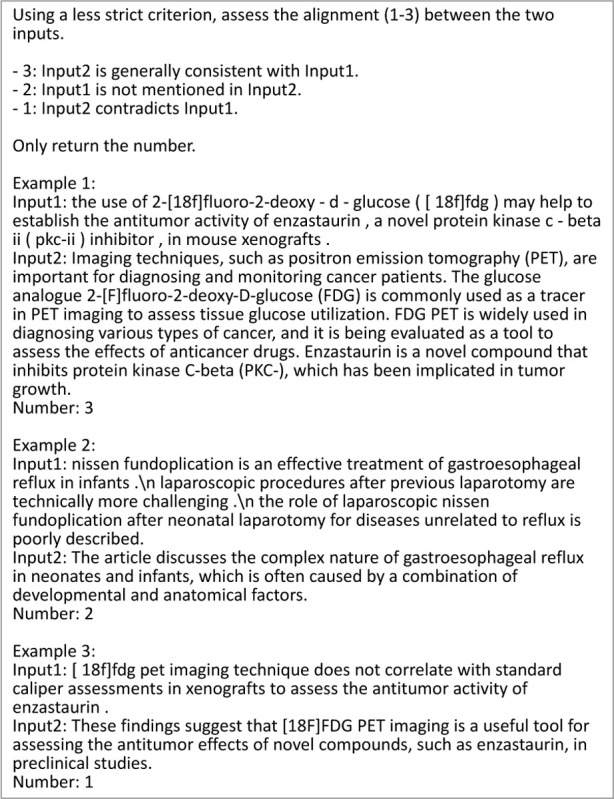
Few-shot prompt for background/conclusion evaluation.

**Figure 7:**
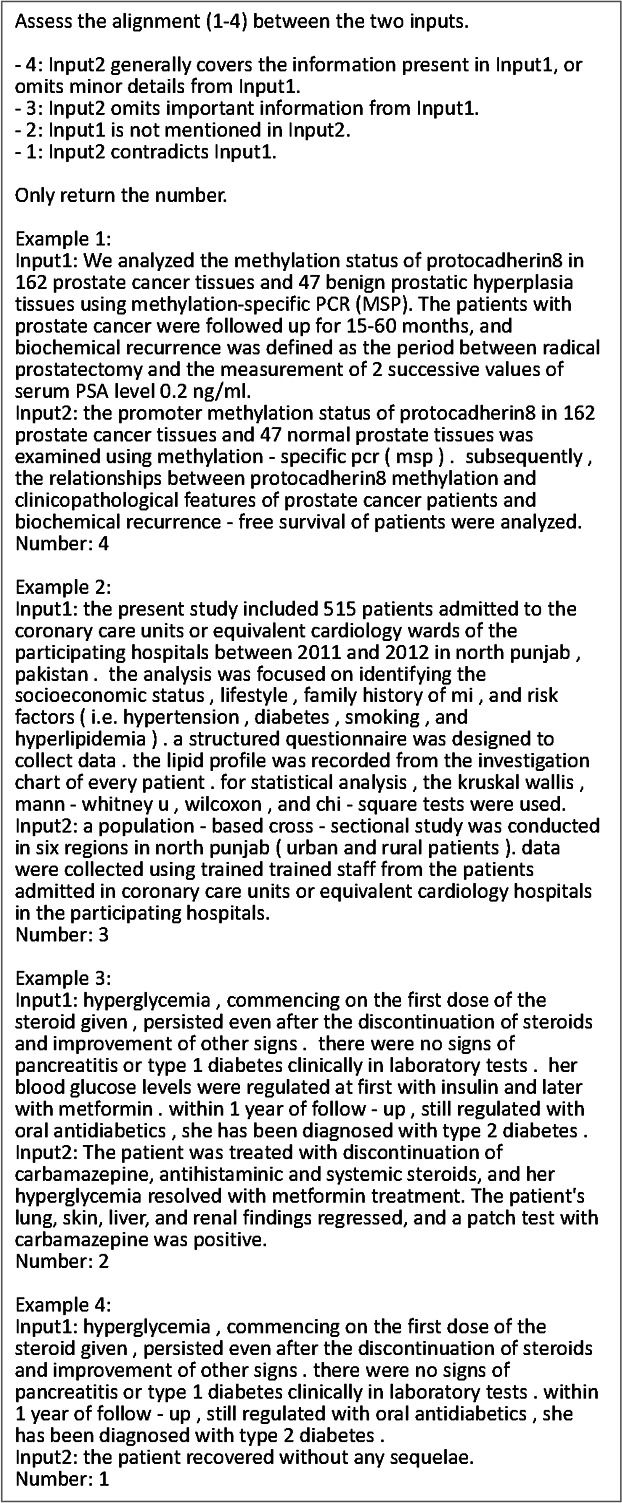
Few-shot prompt for method/result evaluation.

**Figure 8:**
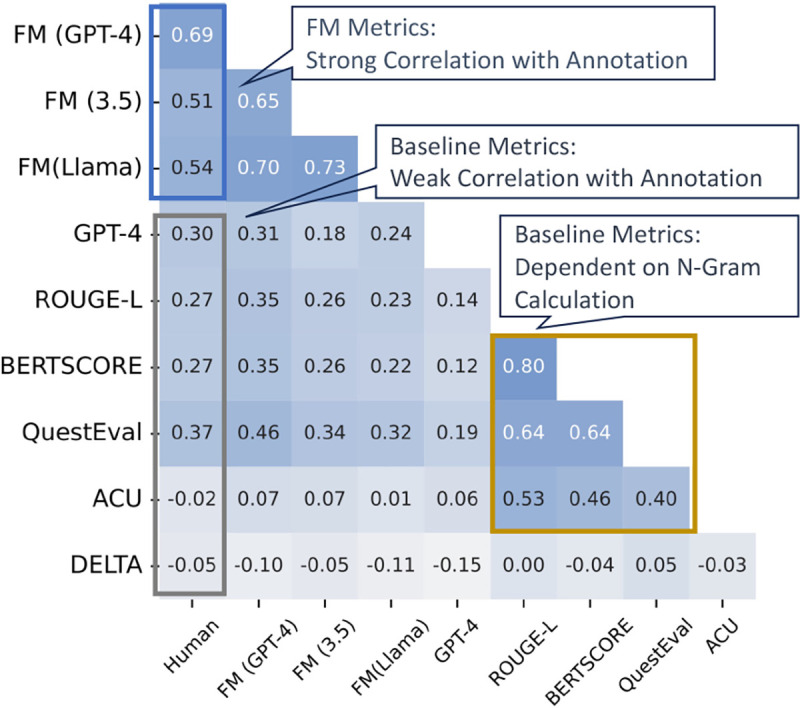
Spearman correlations among metrics within our FM paradigm, LLM-based baseline (GPT-4), and existing evaluation metrics (ROUGE-L, etc) on arXiv dataset.

**Figure 9:**
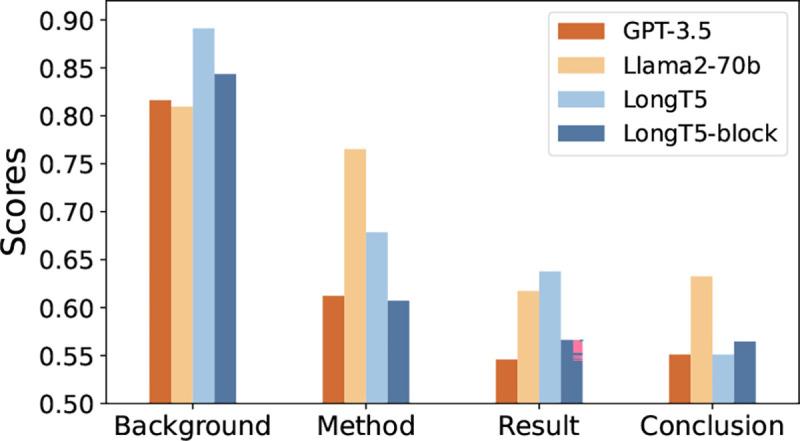
Model performance across four facets.

## A Facet-aware Metric

### A.1 Prompts

In our facet-aware metric, we employ GPT-4 to initially extract information of different aspects within the abstract. The prompt we use is:
What is the background/method/result/conclusion of this work? Extract the segment of the input as the answer. Return the answer in JSON format, where the key is background/method/result/conclusion. If any category is not represented in the input, its value should be left empty.

The evaluation prompt for different facets with in-context samples are shown in Fig. 6 and Fig. 7.

### A.2 Facet Information Extraction Evaluation

We conducted a human evaluation to assess GPT-4’s performance in the facet information extraction task as shown Fig. 5. Generally, GPT-4 exhibits solid performance in extraction tasks, achieving 90% accuracy. However, it does make errors, such as mixing different aspects, omitting certain aspects, and making up information that isn’t present in the input. This issue of generating non-existent information, often referred to as hallucination, is a common phenomenon in LLMs. We are optimistic about the development of more refined LLMs in the future to address these challenges.

## B Comparison of Summarization Systems on arXiv

We show the performance of various summarization systems in different metrics on arXiv in Tab. 4, and the Spearman correlations among metrics within our FM paradigm and existing evaluation metrics on arXiv in Fig. 8.

## C Performance in Different Facets

Beyond the overall evaluation, we show the human evaluation of the models’ performance in various aspects of abstract writing in Fig. 9. Firstly, all models show higher performance in the background aspect, as it often involves just a broad, less detailed overview. In contrast, other aspects demand more precise alignment with the input, leading to generally lower model performance in these areas. Among these three aspects, Llama2 consistently exhibits relatively higher performance. In comparison, GPT-3.5 and other PLMs exhibit weaker performance, especially in formulating conclusions. Specifically, in scenarios where the work’s conclusion deviates from conventional results mentioned in the background, *GPT-3.5 can adhere to the conventions instead of being faithful to the conclusion in the input.* This could be because GPT-3.5 relies more on its internal knowledge base, without thoroughly analyzing the input content. We additionally have a statistical analysis that reveals 34.7% of weak performance cases (where the conclusion score is below 3) in PLM models are due to fluency issues in longer text generation in the last conclusion part.

**Table 4:** Performance of various summarization systems in different metrics on arXiv dataset. **Bold** indicates the best result, while bold denote the second best. Generally, all metrics favor Llama2 and FactSum. Meanwhile, metrics adopting our facet-aware paradigm, including FM(GPT-3.5), FM(GPT-4), and Human, deviate from the existing baselines, often awarding higher scores to Llama2, providing a different perspective on model evaluation.

Model	ROUGE-L	BERTScore	DELTA	QuestEval	ACU	FM(GPT-3.5)	FM(GPT-4)	Human

GPT-3.5	0.2016	0.8339	0.2730	0.1302	0.0495	0.6301	0.6193	0.6556
Llama2	0.2327	0.8371	0.2792	0.1752	0.0000	**0.6611**	**0.6952**	**0.7339**
FactSum	**0.3062**	**0.8661**	**0.3009**	**0.2125**	**0.1605**	0.6578	0.6882	0.7002
BART-Large	0.2220	0.8493	0.2767	0.1479	0.0938	0.5960	0.5850	0.6370
